# Correlation Analysis of *Helicobacter pylori* Infection and Digestive Tract Symptoms in Children and Related Factors of Infection

**DOI:** 10.18502/ijph.v49i10.4694

**Published:** 2020-10

**Authors:** Xiaohong YU, Dongjin FENG, Guangmeng WANG, Zhongmao DONG, Qi ZHOU, Yuan ZHANG

**Affiliations:** Digestive System Department, Xuzhou Children’s Hospital, Xuzhou Medical University, Xuzhou 221000, P.R. China

**Keywords:** *Helicobacter pylori*, Infection, Digestive tract symptoms, Risk factors, Cytokines

## Abstract

**Background:**

To analyze the correlation between *Helicobacter pylori* infection and digestive tract symptoms in children and other related factors, and to explore the risk factors of *H. pylori* infection in children and the expression of inflammatory factors in *H. pylori*-positive and *H. pylori*-negative children.

**Methods:**

Overall, 234 children with *H. pylori* test in Xuzhou Children’s Hospital, Xuzhou Medical University (Xuzhou, China) were enrolled. Among them, 73 children were *H. pylori*-positive and 161 were *H. pylori-*negative. The expression levels of cytokines interleukin-8 (IL-8), interleukin-18 (IL-18) and interferon-γ (IFN-γ) in *H. pylori*-positive and *H. pylori*-negative children were determined by ELISA. The correlation between *H. pylori*-positive and general data, digestive tract symptoms, other clinical symptoms, living habits, eating habits, family history and other related factors was statistically analyzed. Multivariate Logistic regression analysis was used to analyze the independent risk factors of *H. pylori* infection in children.

**Results:**

Family monthly income, inattentive eating, sharing toothbrushes and cups, gnawing fingers, eating fried food, drinking raw water, eating smoked and pickled food, father suffering from gastropathy and mother suffering from gastropathy were independent risk factors for *H. pylori* infection in children. The most common digestive tract symptoms of children with *H. pylori* infection were abdominal pain, accompanied by one or more clinical symptoms. The expression levels of IL-8, IL-18 and IFN-γ in *H. pylori*-positive children were significantly higher than those in *H. pylori*-negative children.

**Conclusion:**

Prevention of *H. pylori* infection in children is helpful for healthy growth of children, and cytokines IL-8, IL-18, IFN-γ have the potential to be used as biomarkers for diagnosis of *H. pylori*-positive children.

## Introduction

*Helicobacter pylori* (*H. pylori*) is a gram-negative bacterium that can live in human stomach and is also one of the most common pathogens in the world. Once infected, it may cause functional dyspepsia, peptic ulcer and even gastric cancer (1–3). According to epidemiological statistics, the infection rate of *H. pylori* is 18.9–87.7%. In 2015, there were about 4.4 billion *H. pylori* -infected patients in the world, with 780,000 cancer cases caused by *H. pylori* (4, 5). *H. pylori* infection mainly lies in childhood, but symptoms begin to appear in adulthood (6). Research on the treatment scheme of *H. pylori* different resistance has become the mainstream treatment choice (7, 8). Therefore, we decided to explore the relevant factors that might affect children’s *H. pylori* infection, to explore the independent risk factors of children’s *H. pylori* infection, and to provide valuable prevention suggestions for children’s *H. pylori* infection.

Interleukin-8 (IL-8) is a kind of proinflammatory chemotactic cytokine with various cell sources, which works on recruiting leukocytes or neutrophils to carry out anti-infection or tissue repair (9,10). The expression of polymorphic IL-8 gene is closely related to *H. pylori* infection and may affect the occurrence of peptic ulcer symptoms in *H. pylori*-positive patients (11). Interleukin-18 (IL-18) is an important cytokine that plays an important role in innate and adaptive immune function and participates in the key innate immune defense process of intracellular infection (12,13). IL-18 can protect mouse models from *H. pylori-*induced asthma and inflammatory bowel disease (14). IL-18 can play a pro-inflammatory role by producing IL-8 from immune cell population, and can also establish a link between immune response and interferon-γ (IFN-γ) by driving polarization of helper T cells and inducing anti-infection natural killer cells (15,16). IFN-γ, a key cytokine produced by inflammatory cells to regulate the development of immune system and related functions, works in immune and tumor monitoring (17, 18). The increase of IFN-γ level plays an important regulatory role in triggering the anti-inflammatory response of helper T cells and preventing *H. pylori* bacterial infection (19). In this study, we investigated IL-8, IL-18 and IFN-γ by detecting their expression levels in *H. pylori*-positive children and *H. pylori*-negative children.

## Data and Methods

### General information

Overall, 234 children tested for *H. pylori* in Xuzhou Children’s Hospital, Xuzhou Medical University, China from Feb 2017 to Feb 2019 were enrolled, including 118 boys and 116 girls, aged 3–15 yr, with an average age of (8.91±3.25) yr, 80 cases at preschool age of 3–6 yr old, 88 cases at school age of 7–12 yr old, 66 cases at puberty of 13–16 yr old, 159 cases from cities and towns, 75 cases from countryside, 73 cases of *H. pylori*-positive and 161 cases of *H. pylori*-negative.

This study was approved by the Ethics Committee of Xuzhou Children’s Hospital, Xuzhou Medical University, and their families were informed and they all signed a fully informed consent.

### Inclusion and exclusion criteria

Inclusion criteria were as follows: those who informed and willing to cooperate with this study; patients without other types of infection; patients with complete clinical data; those who had not received *H. pylori* infection treatment; those who had not undergone surgery for nearly a month. Exclusion criteria were as follows: Bismuth, proton pump inhibitors, H_2_ receptor blockers, antibiotics and other drugs were ingested in the past four weeks; patients comorbid with serious dysfunction of heart, liver, spleen, lung, kidney, etc.; those with communication difficulties; those who were unwilling to sign a fully informed consent.

### Diagnostic criteria for H. pylori infection

*H. pylori* was positive in gastric mucosa tissue culture, and there was a large range of *H. pylori* in gastric mucosa tissue section staining, which was consistent with one diagnosis of *H. pylori* infection. There was a small range of *H. pylori* in gastric mucosa tissue section staining. As a result, 13C urea breath test were positive, serum *H. pylori*-IgG was positive or stool *H. pylori* antigen was positive, and rapid urease test were positive, which was in accordance with the second diagnosis of *H. pylori* infection.

### Observation indicators

Through examining pathological data and designing questionnaires, relevant factors were statistically recorded to carry out correlation analysis and risk factor analysis of *H. pylori*-positive, including general data such as gender, age, place of residence, character, family population, family monthly income. Within six months, whether there were digestive tract symptoms such as reflux, abdominal pain, epigastric pain, nausea and vomiting, hematochezia and peptic ulcer, other clinical symptoms such as recurrent urticaria, anemia, malnutrition, anaphylactoid purpura, anorexia and halitosis, living habits such as inattentive eating, sharing tableware, toothbrushes and cups, keeping pets and gnawing fingers often, eating habits such as eating snacks or fried food, drinking raw water, eating smoked and pickled food, and eating garlic often, family history such as father suffering from gastropathy, mother suffering from gastropathy, and diners suffering from gastropathy.

### Detection methods

Gastric mucosa tissue of subjects was taken out by gastroscopy, incubated with RPMI 1640 medium of 10%FBS at 37 °C for one day, ground evenly, centrifuged at 18,000 r/min for 25 min, and 1 ml of the absorbed supernatant was stored in a freezer at −35 °C. Serum was taken out from the freezer, placed in a refrigerator at 4 °C for dissolution, and then placed at room temperature for complete dissolution. The expression levels of IL-8, IL-18 and IFN-γ in gastric mucosa tissue were detected by ELISA (20), and the detection was carried out in strict accordance with the specifications of human IL-8 ELISA kit, human IL-18 ELISA kit and human IFN-γ ELISA kit (Shanghai Zhenyu Biotechnology Co., Ltd., Shanghai, China, E-EL-H0048km, E-EL-H0253km, E-EL-H0108km). Three kinds of wells were set up: sample, standard and blank wells. Altogether 50 μL of sample to be tested was added to the sample well and 50 μL of standard was added to the standard well, nothing in the blank well. The sample well and the standard well were each added with 100 μL of horse-radish peroxidase labeled detection antibody, sealed and incubated at 37 °C for 60 min. The liquid was poured out, spin-dried and washed 5 times repeatedly. Substrates A and B were fully mixed in a volume of 1:1, 100 μL of substrate mixed solution was added to all wells, the plates were sealed, incubation was carried out at 37 °C for 15 min, and 50 μL of termination solution was added to each well. The absorbance (OD value) at 450 nm of each well was read by an enzyme-labeled analyzer (China Shanghai Chenlian Biotechnology Development Co., Ltd., MB530), and the concentrations of IL-8, IL-18 and IFN-γ were calculated.

### Statistical analysis

The data were processed by GraphPad Prism 6 (GraphPad Software, San Diego, USA). The counting data were expressed by the number of cases/percentage [n(%)], and chi-square test was used for comparison of counting data between groups. The measurement data were expressed by mean±SD, and the comparison of measurement data between the two groups was conducted by *t* test with independent samples. *P<*0.05 was considered to be statistically significant.

## Results

### General information

In the general data of the study subjects, the correlation between *H. pylori* infection and gender was not significant, but the correlation between *H. pylori* infection and age, place of residence, personality, family population and monthly family income was significant (*P<*0.05) (Table 1).

**Table 1:** Correlation between general data and *H. pylori* infection [n(%)]

***Category***	***n***	***H. pylori-positive***	***χ^2^/t***	***P***
Gender			0.112	0.737
Male	118	38 (32.20)		
Female	116	35 (30.17)		
Age (yr)			28.220	<0.001
3–6	80	11 (13.75)		
7–12	88	26 (29.55)		
13–16	66	36 (54.55)		
Place of residence			21.087	<0.001
Cities and towns	159	34 (21.38)		
Countryside	75	39 (52.00)		
Personality			8.639	0.035
Outgoing	124	29 (23.39)		
Introverted	76	28 (36.84)		
Irritable	19	9 (47.37)		
Autistic	15	7 (46.67)		
Number of households			5.833	0.016
(persons)				
≤3	164	59 (35.98)		
＞3	70	14 (20.00)		
Family monthly income			4.880	0.027
(yuan)				
≤5000	94	37 (39.36)		
＞5000	140	36 (25.71)		

### Digestive tract symptoms in children with H. pylori infection

Digestive tract symptoms of children with *H. pylori* infection include reflux, abdominal pain, epigastric pain, nausea and vomiting, hematochezia and peptic ulcer. Among them, abdominal pain accounted for the highest percentage (47.95%), followed by reflux (32.88%), nausea, vomiting and hematochezia accounting for the lowest percentage (2.74%) (Table 2).

**Table 2:** Digestive tract symptoms of children with *H. pylori* infection

***Clinical symptoms***	***Children with H. pylori infection (n=73)***	***Percentage (%)***
Reflux	24	32.88
Abdominal pain	35	47.95
Epigastric pain	11	15.07
Nausea and vomiting	2	2.74
Hematochezia	2	2.74
Peptic ulcer	10	13.70

### Other clinical symptoms of children with H. pylori infection

Children with *H. pylori* infection were also accompanied by recurrent urticaria, anemia, malnutrition, allergic purpura, anorexia, halitosis and other clinical symptoms. Anorexia accounted for the highest percentage (17.81%), followed by halitosis (15.07%), and malnutrition (4.11%) with the lowest percentage. It should be noted that some *H. pylori* children were accompanied by various clinical symptoms (Table 3).

**Table 3:** Other clinical symptoms of children with *H. pylori* infection

***Clinical symptoms***	***Children with H. pylori infection (n=73)***	***Percentage (%)***
Recurrent urticaria	4	5.48
Anemia	9	12.33
Malnutrition	3	4.11
Allergic purpura	5	6.85
Anorexia	13	17.81
Halitosis	11	15.07

### Correlation between infection and children’s living habits

*H. pylori*-infected children had significant correlation with inattentive eating, sharing tableware, sharing toothbrushes and cups, keeping pets and gnawing fingers (*P<*0.05) (Table 4).

**Table 4:** Correlation between *H. pylori* infection and children’s habits

***Living habits***	***n***	***H. pylori-positive***	***χ^2^/t***	***P***
Inattentive eating			7.270	0.007
Yes	98	40 (40.82)		
No	136	33 (24.26)		
Sharing tableware			28.220	<0.001
Yes	80	11 (13.75)		
No	88	26 (29.55)		
Sharing toothbrushes and cups				
Yes	159	34 (21.38)	21.087	<0.001
No	75	39 (52.00)		
Keeping pets				
Yes	159	34 (21.38)	8.639	0.035
No	75	39 (52.00)		
Gnawing fingers			8.639	0.035
Yes	19	9 (47.37)		
No	15	7 (46.67)		

### Correlation between *H. pylori* infection and children’s eating habits

*H. pylori* infection was significantly correlated with eating habits such as eating snacks, or fried food, drinking raw water, eating smoked and pickled foods, and eating garlic often (*P<*0.05), but not with eating vegetables raw (Table 5).

**Table 5:** Correlation between *H. pylori* infection and children’s eating habits

***Eating habits***	***n***	***H. pylori-positive***	***χ^2^/t***	***P***
Eating snacks often			8.165	0.004
Yes	115	46 (40.00)		
No	119	27 (22.69)		
Eating fried food often			9.123	0.003
Yes	110	45 (40.91)		
No	124	28 (22.58)		
Drinking raw water often			6.692	0.010
Yes	84	35 (41.67)		
No	150	38 (25.33)		
Eating vegetables raw often			2.870	0.090
Yes	75	29 (38.67)		
No	159	44 (27.67)		
Eating smoked and pickled food often			5.312	0.021
Yes	72	30 (41.67)		
No	162	43 (26.54)		
Eating garlic often				
Yes	65	27 (41.54)	4.485	0.034
No	169	46 (27.22)		

### Correlation between *H. pylori* infection and children’s family history

*H. pylori* infection had a significant correlation with the family history of children such as father suffering from gastropathy, mother suffering from gastropathy, and diners suffering from gastropathy (*P<*0.05) (Table 6).

**Table 6:** Correlation between infection and children’s family history

***Eating habits***	***n***	***H. pylori-positive***	***χ^2^/t***	***P***
Father suffered from gastropathy			5.216	0.022
Yes	69	28 (40.58)		
No	175	45 (25.71)		
Mother suffered from gastropathy			4.485	0.034
Yes	65	27 (41.54)		
No	169	46 (27.22)		
Diners suffered from gastropathy			10.791	0.010
Yes	80	36 (45.00)		
No	154	37 (24.03)		

### Multivariate Logistic regression analysis on influencing factors of *H. pylori* infection in children

Monthly family income (*P=*0.041), inattentive eating (*P=*0.010), sharing toothbrushes and cups (*P=*0.001), gnawing fingers (*P=*0.043), eating fried food often (*P=*0.048), drinking raw water often (*P=*0.045), eating smoked and pickled foods often (*P=*0.020), father suffering from gastropathy (*P=*0.034), and mother suffering from gastropathy (*P=*0.026) were independent risk factors for *H. pylori* infection in children (Table 7).

**Table 7:** Multivariate Logistic regression analysis on influence of *H. pylori* infection in children

***Variable***	***B***	***S.E***	***Wals***	***P***	***OR***	***95% CI***
Monthly household income	1.304	0.238	8.789	0.041	2.106	1.496–3.301
Inattentive eating	1.458	0.190	15.599	0.010	3.124	1.643–5.018
Sharing toothbrushes and cups	1.513	0.157	18.322	0.001	1.897	2.029–6.986
Gnawing fingers	1.285	0.215	6.693	0.043	1.838	1.372–2.782
Eating fried food often	1.221	0.243	4.589	0.048	1.664	1.327–2.940
Drinking raw water often	0.534	0.246	4.645	0.045	1.709	1.057–2.769
Eating smoked and pickled food often	0.967	0.295	10.584	0.020	2.621	1.461–4.693
Father suffered from gastropathy	1.306	0.219	8.337	0.034	2.649	1.385–3.976
Mother suffered from gastropathy	1.302	0.226	10.098	0.026	2.810	1.503–5.028

### Expression levels of IL-8, IL-18 and IFN-γ in *H. pylori*-positive and *H. pylori*-negative children

**Fig. 1:**
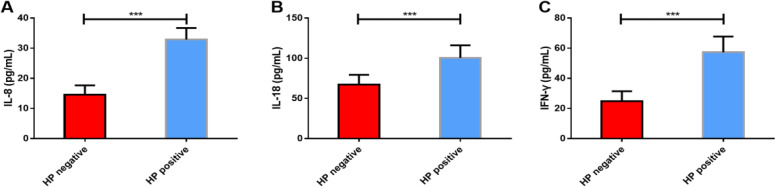
Expression levels of IL-8, IL-18 and IFN-γ in *H. pylori*-positive and *H. pylori*-negative children (A) IL-8 in *H. pylori*-positive children was significantly higher than that in *H. pylori*-negative children. (B) IL-18 in *H. pylori*-positive children was significantly higher than that in *H. pylori*-negative children. (C) IFN-γ in *H. pylori*-positive children was significantly higher than that in *H. pylori*-negative children. Note: ^***^ *P<*0.001

The expression levels of IL-8, IL-18, IFN-γ in *H. pylori*-negative children were significantly lower than those in *H. pylori*-positive children (*P<*0.05) (Fig. 1).

## Discussion

Among the relevant symptoms of *H. pylori* infection in children, the typical digestive tract symptom is abdominal pain, and other common clinical symptoms are anorexia, halitosis, malnutrition, etc. Studies have shown that anorexia caused by *H. pylori* infection is related to activation of hypothalamic cavitation cytotoxin, while halitosis caused by *H. pylori* infection may be related to increase of H2S content caused by *H. pylori* decay (21, 22). In the research on the relationship between *H. pylori* infection and digestive tract diseases, *H. pylori* might cause gastritis, peptic ulcer, malignant lymphoma and even diseases outside the digestive tract, eventually leading to malnutrition in children, manifested by short stature, which suggested that preventing *H. pylori* infection was important for children’s development (23). Therefore, it is of great significance for the improvement of children’s quality of life and healthy growth to find out the relevant risk factors of *H. pylori* infection and propose preventive measures with reference significance.

At present, more and more scholars have carried out relevant researches on the relevant factors affecting children’s *H. pylori* infection. Based on the study of risk factors of *H. pylori* infection in children, factors such as age over 10 yr, low income, too many family members, bed sharing and two parents infected with *H. pylori* were positively correlated with *H. pylori* infection in children, suggesting that improving social economy and quality of life might prevent *H. pylori* infection in children (24). Low monthly income and low educational level were risk factors for *H. pylori* infection in children, suggesting that besides improving social economy, improvement of educational level also played a role in preventing *H. pylori* infection in children (25). In a study on risk factors of *H. pylori* infection in asymptomatic children, factors such as hand disinfection, eating alone, higher education level of mother, higher quality of life than average and living in cities and towns might protect children from *H. pylori* infection, suggesting that healthy living habits, high quality of life and education level, and city life had certain positive effects on protecting children from *H. pylori* infection (26). The family history of gastric cancer meant a higher *H. pylori* infection rate, which might be related to the expression of more virulent genes caused by more frequent colonization of *H. pylori* strains by relatives of gastric cancer (27). It should be emphasized that artificially controlling *H. pylori* infection could reduce the correlation between the family history and the risk of gastric cancer, indicating that the family history of gastric cancer was not the main cause of *H. pylori* infection. In this study, the key behaviors that children or their families could control to reduce the risk of *H. pylori* infection were to increase family monthly income, concentrate on eating, use personal toothbrushes and cups, correct the habit of gnawing fingers, eat less or preferably no fried food and smoked or pickled food, and do not drink raw water, which was also the health strategy we advocated to prevent *H. pylori* infection in children.

Cytokines IL-8, IL-18 and IFN-γ also participated in the regulation of *H. pylori* infection. As to the relationship between serum IL-8 and *H. pylori* infection in gastric cancer patients, the expression of serum IL-8 in *H. pylori*-positive patients was significantly higher than that in negative controls and was significantly positively correlated with *H. pylori* infection, suggesting that IL-8 might participate in the occurrence and progress of *H. pylori* infection(28). High levels of IL-8 in serum were associated with *H. pylori* infection and recurrent abdominal pain in children (29). In the in vitro study on the relationship between IL-18 and *H. pylori* infection (30), abnormal secretion of IL-18 caused by *H. pylori* infection could in turn enhance the host’s stress response to *H. pylori* infection. IFN-γ had a higher level of expression in *H. pylori*-infected children and was significantly associated with chronic antral inflammation (31). In our study, the expression levels of cytokines IL-8, IL-18 and IFN-γ in gastric mucosa tissues in children with *H. pylori*-positive were significantly higher than those in negative controls, suggesting that they might become biomarkers for diagnosing *H. pylori*-positive, but the specific diagnostic value would not be analyzed for the time being in this study.

Although this study confirmed that healthy living and eating habits, improving family economic level had certain preventive effects on children’s *H. pylori* infection, and IL-8, IL-18, IFN-γ were highly expressed in children’s *H. pylori*-positive, there was still some room for improvement. First of all, we could expand the sample, extract relevant data from the database for large sample analysis, and improve the reliability of the research. Second, we could supplement the diagnostic value of the above cytokines for *H. pylori-*positive. Further, basic experiments could be conducted to explore the specific regulatory mechanism of the above cytokines on *H. pylori* infection. We need to perfect these points and further supplement them in future research.

## Conclusion

Prevention of *H. pylori* infection in children is helpful for healthy growth of children, and cytokines IL-8, IL-18, IFN-γ have the potential to be used as biomarkers for diagnosis of *H. pylori-*positive children.

## Ethical considerations

Ethical issues (Including plagiarism, informed consent, misconduct, data fabrication and/or falsification, double publication and/or submission, redundancy, etc.) have been completely observed by the authors.
